# The Book World of Medicine and Science

**Published:** 1893-08-26

**Authors:** 


### THE BOOK WORLD OF MEDICINE AND SCIENCE.

### BOOK REVIEWS.

### HOSPITALS OF THE WORLD*—OUT PATIENTS.

The institution of out-patients' department in all the large hospitals, which promised in the beginning to be a welcome relief in the constant pinch for accommodation and an untold boon to the neighbouring poor, has proved in its development to be a serious bar to hospital and general medical progress. It has encouraged among persons of almost every class a theory that all are entitled to free medical relief, and the effects of this theory upon the profession at large are increasingly disastrous, while the recipients of the aid cannot fail to be injured even more seriously by the weakening of a just sense of independence.

As early as the year 1742 out-patients were treated at the London Hospital to the number of 2,188 in eighteen months. It was usual for them to attend and return thanks before the committee after their term of treatment was over, and it is probable that the arrangements were not such as to encourage those not actually in need to claim assistance.

Since that time the number of out-patients gratuitously treated has swollen beyond all proportion. The following statistics, showing the number of out-patients per 1,000 in twelve large towns must awake in the most incredulous a sense of there being something wrong somewhere :—

| | Population. | Out-patients. | Population per 1000. |
|---|---|---|---|
| Dublin ............ | 353,082 ... | 162,164 ... | 459 |
| Liverpool ......... | 517,116 ... | 198,378 ... | 383 |
| | Population. | Out-patients. | Proportion per 1000. |
| Edinburgh......... | 261,970 ... | 95,535 ... | 365 |
| Birmingham ...... | 429,906 ... | 144,559 ... | 336 |
| Manchester ...... | 506,469 ... | 146,298 ... | 288 |
| London ........... | 4,221,452 ... | 1,158,026 ... | 274 |
| Glasgow ........... | 567,143 ... | 85,839 ... | 151 |
| Norwich............ | 101,316 ... | 10,577 ... | 104 |
| Cardiff ............ | 130,283 ... | 11,058 ... | 85 |
| Halifax ............ | 83,109 ... | 4,198 ... | 50 |
| Portsmouth ...... | 160,128 ... | 6,863 ... | 42 |
| Oldham ............ | 132,000 ... | 3,711 ... | 28 |

* "Hospitals and Asylums of the World." By Henry C. Burdett. London: The Scientific Press, 428, Strand, W.C. 4 Vols. and Portfolio of Plans, £8 8s.

To understand the full significance of these figures, it must be remembered that a very large proportion (Mr. Burdett estimates 50 per cent.) must be added to them in each town of those in receipt of free medical aid from the poor law infirmaries and free dispensaries. "Who will venture to contend that one in every two of the population, or one in every two and a half, or even one in every three persons in a great centre of population, can under any conceivable circumstances require, or be justified in receiving, free out-patient relief at the hospitals?" Moreover, the disproportion between such towns as Manchester and Oldham, both wealthy, in the same district, and given up to much the same trades, points to the probability of a very large amount of needlessly gratuitous doctoring. In country districts where long distances must be travelled to obtain even poor law medical aid, labourers supporting themselves and families on considerably less than £1 a week commonly find the money to pay a village doctor. In towns this is so unusual, that a cabman applying to the Relief Committee of his church for assistance in a dangerous illness was held to be disqualified

**The Hospital Advertiser.**

THE BOOK WORLD OF MEDICINE AND SCIENCE—*continued.*

because he was found to be bravely paying the doctor 2s. a visit out of a small reserve fund of savings.

It has been seen that the existing out-patient system tends to pauperise the population and damage the medical profession. There is yet another aspect to be considered. "It is also a question for the medical profession how far, if at all, any medical man of ability and standing is justified in devoting a large amount of his time to out-patient work under conditions which render that work perfunctory, incomplete, and at times ludicrously inadequate, if a due regard be paid to the decencies of treatment and a reasonable amount of time be given to each case with a view to an exhaustive examination and a correct diagnosis."

It is easier to recognise the evil than to take even one step towards removing it. More than one attempt has been made to improve matters, but so far without result. In 1871 a Commission under Sir William Ferguson issued, after an elaborate investigation of facts, the following recommendations:—

1. An improved administration of poor law medical relief.
2. A placing of all free dispensaries under the control of the poor law authorities to secure a proper system of inquiry.
3. The establishment of a system of provident dispensaries.
4. The curtailing of the present unrestricted system of gratuitous relief at the general and special hospitals, partly by the selection of cases possessing special clinical interest, and partly by the exclusion of persons able to pay.
5. The abolition of all payments for medicine, and free medical advice from out-patients.
6. The payment of the medical staff engaged in out-patient work.

The Birmingham Hospital Reform Inquiry Committee, which began its meetings in 1889, arrived at much the same conclusions. In addition the committee recommended the formation of a general council on which all the hospitals should be represented, together with the corporation of the city and the poor law guardians.

The drastic and highly original plan is suggested in "The Hospitals of the World" of closing all the out-patient departments for six months, with a view to a complete reorganisation. Such is the ease with which medical aid is procured, in London at any rate, that the author is probably justified in his supposition that "It would be found that the majority of the cases were able to find the relief they required, either at the dispensaries (poor law and voluntary), or at the hands of the private medical practitioners who practise in the neighbourhoods where these people reside." It is, of course, understood that urgent medical and accident cases would continue to be dealt with at the surgeries of the hospitals But, pending the execution of this interesting experiment, which may be expected to take place some time in the Greek Kalends, the author urges the introduction of the following important reforms. And first as regards the payment of the medical staff at hospitals. "A close study of all the issues involved leads us to the conclusion that no effectual remedy will be possible until every member of the medical profession attached to the voluntary hospitals of this country receives remuneration for his services." The lay-managers are inevitably hampered in all efforts at reform by the prerogatives of a medical staff entitled to special consideration on the ground of the eleemosynary service rendered to the hospitals. To the objection that the

**The Hospital Advertiser.**

THE BOOK WORLD OF MEDICINE AND SCIENCE—*continued.*

hospitals cannot afford to pay the medical staff, the author urges "an inquiry by a joint committee of medical men and laymen attached to each hospital into the whole question of medical services as it is at present rendered, with the object of securing such a rearrangement of the system now in force as will secure that the funds of the hospitals shall not be trenched upon by the medical school, and that so it shall be possible to offer adequate, or at least reasonable, remuneration to all members of the medical staff."

It is also urged that some restriction should be placed on the use of letters by subscribers who are employers of labour, since these subscribers, posing as generous benefactors of the hospital, often receive out of the institution in actual value a sum many times greater than the annual contribution. The system advocated for ensuring just contributions from the recipients of the relief and for discouraging unnecessary resort to the hospital is admirable. "An attempt should be made to induce every workshop to establish a box, and to put into it a weekly subscription from each of the employés, together with an adequate contribution from the masters. The money so obtained to be used, in the first instance, to pay the fees of the local medical practitioner, to whom the workman or his family would be first referred by the foreman, and who would attend them in all ordinary illnesses. If it was necessary for a case to go to a hospital, then a letter of introduction would be sent to the hospital with the patient from the workshop. All the money which the box contains at the close of the year to be sent as a free gift to the Central Hospital Fund. Thus the interests of the artisans, the hospitals, and the general practitioners of medicine would be alike protected."

If this system could take root and extend among shop assistants, clerks, elementary teachers, &c., of both sexes, most of whom are quite as awkwardly situated with regard to doctors' fees as the artisan, there is reason to hope that the out-patient department at the hospitals would begin to assume something like due proportions.

THE BOOK WORLD OF MEDICINE AND SCIENCE—*continued.*

us! We look for him always to be brilliant, and although parts of his article are terse, original, and graphic, yet it would be an error to speak of it as quite worthy of the great reputation Dr. Billings has won. The opening paragraphs are by no means happy. They consist of a partly humorous defence of the practitioner who tries to prevent disease, although at first sight he may "seem to be killing the goose that lays the golden egg." To put the money advantages of such conduct in the forefront of our article on "Hygiene" is not worthy. Dr. Pepper follows with articles on typhoid fever, typhus, relapsing fever, &c., and Drs. Whittaker and Gilman Thomson write on the other acute specific fevers. We note that Dr. Pepper speaks highly of Brand's treatment of typhoid by the cold bath. The chapters on the specific fevers are certainly good. The subject is dealt with systematically, fully, and practically. The articles are not exhaustive, but amply full enough for the average reader, they are well up to date, and while free use is made of the writings of others, the author's own experience is the foundation on which the teaching mainly rests, and this makes them of real practical value. Of course, the reader finds the fruit of modern research as to the pathology of these diseases and their relation to micro-organisms—this part is well done. Diseases of the nervous system are dealt with Dr. Osler and Dr. Horatio C. Wood. Dr. Osler is well-known as an accomplished physician and able writer. His articles are some of the best in the volume. If the second volume is as good as the first this text-book will be highly creditable to American teachers, and we fancy it will find readers on this side of the Atlantic as well as in the United States.

The Birmingham Medical Review. August, 1893.

This number contains one original article by Dr. Edwin Rickards on infectious diseases and their treatment by vaccines. Among the cases reported is a good one of Myxodema greatly benfited by powders of Thyroid extract, a case of Primary Carcinoma of the lung which ran a very rapid course, and three cases of Gangrenous Bowel in Strangulated Hernia. The "Periscope" is devoted to surgical diseases of the genito-urinary organs.

Oxygen Sewage Purification.

Mr. J. C. Melliss, M.Inst., has published a favourable report to the directors of the Oxygen Sewage Purificaction Company on the working of their system at the Central Criminal Lunatic Asylum, Dundrum. The system consists in allowing the sewage to flow in succession through three deep tanks, entering each at the bottom and escaping at the top. In the first tank the bulk of the solid matter in suspension falls to the bottom; in the second tank precipitation by manganate of soda and sulphate of alumina is effected, and in the third tank a further process of oxydation is carried out by the addition of nitrate of soda. Mr. Melliss reports that the process is inexpensive, inoffensive, and effectual.

